# Dietary Intake after Weight Loss and the Risk of Weight Regain: Macronutrient Composition and Inflammatory Properties of the Diet

**DOI:** 10.3390/nu9111205

**Published:** 2017-11-02

**Authors:** Harry Freitag Luglio Muhammad, Roel G. Vink, Nadia J. T. Roumans, Laura A. J. Arkenbosch, Edwin C. Mariman, Marleen A. van Baak

**Affiliations:** 1 Department of Nutrition and Health, Faculty of Medicine, Universitas Gadjah Mada, Jalan Farmako, Sekip Utara, Yogyakarta 55281, Indonesia; 2 Department of Human Biology, NUTRIM School of Nutrition and Translational Research in Metabolism, Maastricht University Medical Centre, 6200MD Maastricht, The Netherlands; r.vink@maastrichtuniversity.nl (R.G.V.); n.roumans@maastichtuniversity.nl (N.J.T.R.); lauraarkenbosch@hotmail.com (L.A.J.A.); e.mariman@maastrichtuniversity.nl (E.C.M.); m.vanbaak@maastrichtuniversity.nl (M.A.v.B.)

**Keywords:** obesity, weight regain, macronutrient composition, inflammation

## Abstract

Weight regain after successful weight loss is a big problem in obesity management. This study aimed to investigate whether weight regain after a weight loss period is correlated with the macronutrient composition and/or the inflammatory index of the diet during that period. Sixty one overweight and obese adults participated in this experimental study. Subjects lost approximately 10% of their initial weight by means of very low-calorie diet for five weeks, or a low calorie diet for 12 weeks. After that, subjects in both groups followed a strict weight maintenance diet based on individual needs for four weeks, which was followed by a nine-month weight maintenance period without dietary counseling. Anthropometrics and dietary intake data were recorded before weight loss (baseline) and during the weight maintenance period. On average, participants regained approximately half of their lost weight. We found no evidence that macronutrient composition during the weight maintenance period was associated with weight regain. The dietary inflammatory index (*r* = 0.304, *p* = 0.032) was positively correlated with weight regain and remained significant after correction for physical activity (*r* = 0.287, *p* = 0.045). Our data suggest that the inflammatory properties of diet play a role in weight regain after weight loss in overweight and obese adults.

## 1. Introduction

It has been estimated that a total of 107.7 million children and 603.7 million adults worldwide were obese in 2015 [1], and this state of over-nutrition was responsible for an increasing economic and health burden [1,2]. Effective actions to prevent the increasing rate of obesity and to treat those who already are obese are required. Lifestyle-based weight loss programs, which include an energy-restricted diet and increased physical activity, are by far the most commonly used weight loss methods. Effective lifestyle-based weight loss interventions have been developed in the last few decades, but maintaining the attained weight loss is not an easy task [3]. Studies have shown that overweight and obese individuals who lost weight through lifestyle modification are prone to weight regain [4,5,6]. 

Several theories have been proposed to explain this weight regain in which biologic and behavioral factors play an important role [7]. Reduction in basal and activity-related energy expenditure, changes in hunger- and satiety-associated hormone production, and an increase in fat cell stress in response to fat mass reduction have been suggested as potential factors associated with the tendency towards gaining weight after weight loss [7,8]. In addition, several eating-related behavioral factors, such as ability to control over-eating, binge eating and eating as a response to negative emotions have also been suggested to increase the risk of weight regain [9].

Several studies have been conducted to evaluate whether dietary factors are associated with weight regain. Several studies found that higher dietary protein content after weight loss was associated with less weight regain [10,11,12,13,14]. However, not all studies support this notion [15,16]. The role of other macronutrients in the prevention of weight regain has been less well studied.

Dietary intake not only has a direct impact on body weight, but may also have indirect effects that are important for body weight regulation. We have previously shown that weight regain after weight loss was correlated with the expression of genes related to adipose tissue stress and inflammation [17,18]. Because inflammation is also influenced by the dietary pattern [19], the inflammatory properties of the diet might play role in weight regain.

Therefore, this study aimed to investigate the influence of the macronutrient composition and inflammatory properties of the diet on weight regain during a weight maintenance period after weight loss of overweight and obese individuals. To analyze the correlation between the inflammatory properties of diet and weight regain we calculated the dietary inflammatory index (DII). The DII consists of a compilation of effects of intake of specific nutrients that have been shown to change inflammatory parameters in the blood, and provides a quantitative estimate of the inflammatory tendency of an individual’s diet [19,20,21,22]. In addition, we analyzed the association between each individual component of the dietary inflammatory index and weight regain. 

## 2. Methods

### 2.1. Subjects

Male (*n* = 27, 45.8%) and female (*n* = 30, 54.2%) Caucasian adults with overweight and obesity participated in this study. The recruitment process was done through advertisements. The inclusion criteria were body mass index more than 28 kg/m^2^ with stable body weight in the past 2 months prior to the study. Those who had a current or previous history of cardiovascular disease, type 2 diabetes mellitus, liver or kidney disease, used medication that influences body weight regulation, were pregnant, smoking, or had marked alcohol consumption were excluded from the study. Subjects with elevated fasting glucose (>6.1 mmol/L), total cholesterol (>7.0 mmol/L) or triacylglycerol (>3.0 mmol/L) concentrations, or blood pressure (>160/100 mmHg) prior to the intervention were also excluded. A total of 61 subjects started this study and provided their written informed consent before participation. The study was performed according to the Declaration of Helsinki and was approved by the Medical Ethics Committee of Maastricht University Medical Centre. This trial is registered at www.clinicaltrials.gov as NCT01559415. This study is a secondary analysis of a study of which the design and methods have been described in detail before [23].

### 2.2. The Dietary Intervention Program

The dietary intervention program was composed of three periods: weight loss period (WL), weight stable period (WS) and follow-up period (FU). The weight maintenance (WM) period is a combination of the weight stable and follow-up periods. During WL, subjects were divided into two groups: a low calorie diet (LCD, 1250 kcal/day) group and a very low calorie diet (VLCD, 500 kcal/day) group. The LCD group replaced one meal by a meal replacement (Modifast; Nutrition et Sante Benelux, Breda, The Netherlands). The other two meals were prepared by the participants themselves based on meal plans designed by a dietitian, based on the Dutch national dietary guidelines, and they consumed three in-between meal snacks. The VLCD group consumed three meal replacements per day with additional 100 mL instant broth drinks per day and an unrestricted amount of low calorie vegetables. The length of the WL period was 12 weeks in the LCD group and 5 weeks in the VLCD group in order to achieve a similar 10% weight reduction in both groups. After the WL period, subjects in both groups followed a strictly balanced diet based on their individual energy requirements for 4 weeks (WS period) according to the Dutch national dietary guidelines [24]. During the follow-up period of 9 months, subjects were advised to maintain their body weight, but they no longer received dietary consultation and were free to choose their diet. 

### 2.3. Measurements

**Figure 1:**
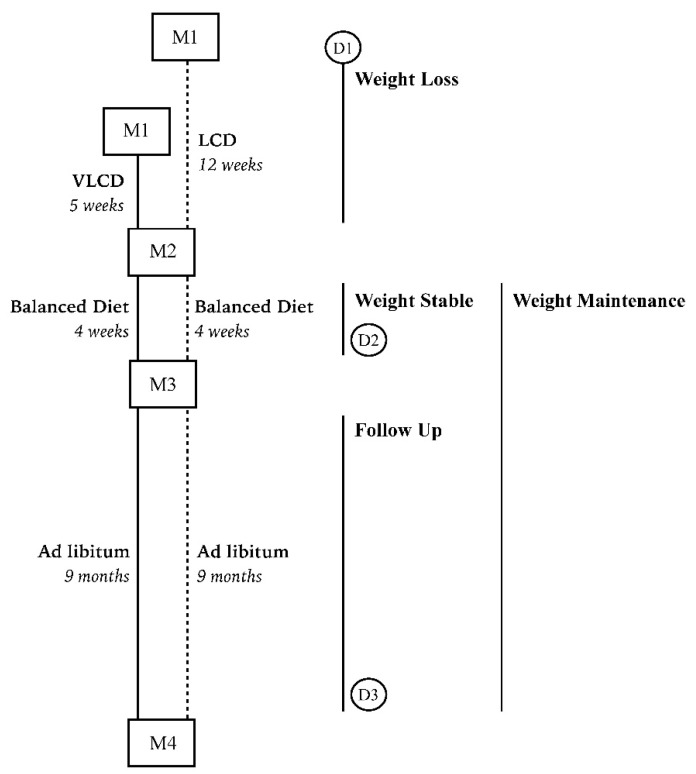
Schematic representation of study design and measurements. Anthropometric measurements M1: before intervention; M2: end of weight loss period; M3: end of weight stable period; M4: end of follow up period. Weight loss (WL) = M2 − M1; weight regain (WR) = M4 − M3. Baseline diet = D1, weight maintenance (WM) diet = average of D2 and D3. Physical activity was measured during M1, M2, M3 and M4.

Anthropometric measurements were done at 4 time points: before the intervention (M1), at the end of WL (M2), at the end of WS (M3) and at the end of FU (M4) (Figure 1). Weight regain was calculated as body weight at M4 minus body weight at M3. Anthropometric measurements were obtained successfully at M1, M2 and M3 in all subjects but only 55 (96.5%) subjects were measured at M4 (Supplement 1). 

Height was measured using a stadiometer (Seca, Hamburg, Germany). Body weight was measured using a digital weighing scale (precision 0.1 kg) (Seca, Hamburg, Germany). Waist circumference was measured above the umbilicus and hip circumference was measured at the widest part of the buttocks. Percent body fat mass was determined by air displacement plethysmography (BodPod, Cosmed, Italy). 

Measurements of dietary intake were conducted at 3 time points: before the weight loss intervention (D1), at the end of WS (D2) and at the end of FU (D3) (Figure 1). At each time point, 3-day food diaries (including 1 non-working day) were collected. All foods reported were linked to the 2011 Dutch food consumption table (NEVO online version 2011, RIVM, Bilthoven, The Netherlands) and nutrient intakes were calculated. Some of the dietary intakes and physical activity differed significantly between D2 and D3 (Supplement 2). However, because we do not know the time course of the changes from D2 to D3, we have assumed that the changes were gradual and the mean of D2 and D3 would best reflect the overall level during the follow-up period. Thus, the nutrient intake values obtained at D2 and D3 were averaged to reflect dietary intake over the weight maintenance (WM) period. Nutrients intakes were corrected for total energy intake [25] and a dietary inflammatory index was calculated according to Tabung et al. [26], based on the work of Shivappa et al. [20]. The calculation of the dietary inflammatory index was based on 27 nutrients including total energy, protein, carbohydrate, total fat, saturated fat, trans fat, mono-unsaturated fatty acid, poly-unsaturated fatty acid, omega-3 fatty acid, omega-6 fatty acid, cholesterol, fiber, alcohol, magnesium, iron, selenium, zinc, vitamin A, vitamin C, vitamin D, vitamin E, thiamin, riboflavin, vitamin B6, vitamin B12, folate and niacin, because no data were available for the other dietary factors included by Shivappa et al. [20] in their dietary inflammation index. Data on dietary intake during weight maintenance were obtained in 52 subjects (91.2%).

Subjects’ total physical activity at baseline (M1), during the weight loss period (M2) and during weight maintenance period (M3 and M4) (Figure 1) was calculated using the sum score of occupational, leisure time and sports activity obtained from the Baecke questionnaire for habitual physical activity [27]. 

### 2.4. Data Analysis

Statistical analysis was performed with SPSS for Macintosh, Version 21 (Chicago, IL, USA). Data are presented as mean ± standard error of the mean. Since no statistically significant differences were found between the VLCD and LCD groups, groups were combined for the correlation analyses. An independent *t*-test was used when comparing data on anthropometric, dietary and physical activity differences between the LCD and VLCD groups before and after the weight loss intervention. Paired *t*-tests were used when analyzing changes (anthropometric and dietary intake) within a group over a specific period of intervention. Wilcoxon signed ranks were used for non-normally distributed data. The correlation between nutrient intake, DII and weight regain was done using Pearson correlation tests for normally distributed data, and Spearman correlation tests for non-normally distributed data. Normality tests were performed using the Kolmogorov Smirnov normality test. In addition, partial correlations with correction for physical activity were determined. The analyses were considered statistically significant when *p* < 0.05 (2-tailed). 

## 3. Results

**Figure 2:**
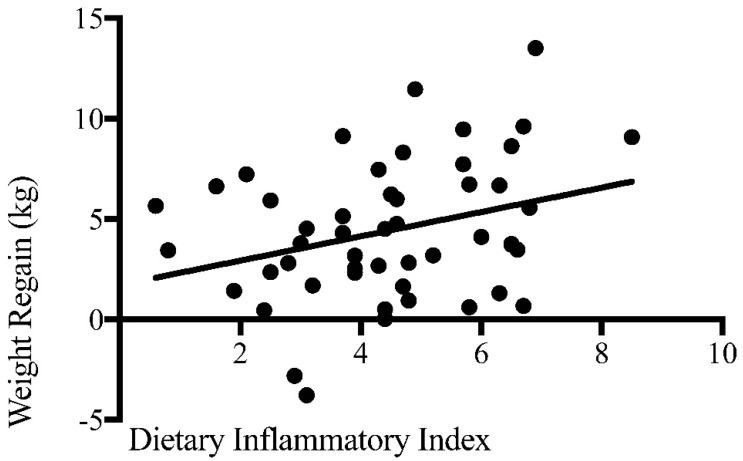
The correlation between dietary inflammatory index and weight regain.

A total of 61 obese subjects were initially recruited in this study, but four subjects withdrew because of health and personal circumstances not related to the study. Data on anthropometric measures are presented in Table 1. Gender was equally distributed (LCD group male = 48.3%, VLCD group male = 46.4%). There were no differences in body weight and other anthropometric measures at any time point (M1, M2, M3 and M4) between subjects in LCD and VLCD groups (Supplement 1). Subjects significantly lost weight at the end of the weight loss period (M2 vs. M1) and gained weight at the end of follow up period (M4 vs. M3) (Table 1). Weight regain varied between −3.8 kg and +13.5 kg (see also Figure 2). 

There were no differences in energy intake and dietary composition between the LCD and VLCD group before and during WM (all *p* > 0.05) (Supplement 2) (Table 2). Subjects in both groups had a significantly lower total energy intake during WM (*p* < 0.001) compared to their initial energy intake. Protein and carbohydrate intake (as percent of total energy intake) increased (all *p* < 0.05) with no change in % energy from sugar consumption, whereas percentage of energy from fat was reduced, mainly due to a reduction in saturated fat (all *p* < 0.05). Alcohol consumption (expressed as g/1000 kcal) did not change significantly over the intervention period. The intake of fiber and several micronutrients (niacin, riboflavin, vitamin B6, folate, vitamin C, vitamin A, zinc, selenium, iron and magnesium), expressed as g or mg/1000 kcal, was increased during the weight maintenance period. In addition, the dietary inflammatory index during the weight maintenance period was lower than the baseline (*p* < 0.001). There was no change in physical activity (*p* = 0.437) over the intervention period.

The correlations between macronutrients, the dietary inflammatory index and its components, and weight regain are shown in Table 3. No significant correlations with the intake of the various macronutrients were found. The dietary inflammatory index and total energy intake were positively correlated with weight regain, while magnesium, riboflavin and folate intake were negatively correlated with weight regain. The scatter plot of the correlation between the dietary inflammatory index and weight regain can be seen in Figure 2. Gender (*r* = 0.036, *p* = 0.794), body mass index (*r* = −0.106, *p* = 0.439), age (*r* = 0.187, *p* = 0.171) and initial weight loss (*r* = 0.008, *p* = 0.955) were not correlated with weight regain, while physical activity (*r* = −0.278, *p* = 0.040) was negatively correlated. Therefore, partial correlation analyses were conducted to evaluate the correlations between dietary intake, dietary inflammatory index and weight regain, independent of physical activity. Energy and riboflavin intake, and the dietary inflammatory index remained significantly correlated with weight regain, but not magnesium and folate.

## 4. Discussion

This experimental study evaluated the association of dietary intake with weight regain during a period of intended weight maintenance after successful weight loss. Subjects reduced their energy intake following a weight loss period, with no significant differences between the groups that had attained their weight loss within five or 12 weeks. The macronutrient composition of the weight maintenance diet was not associated with weight regain. In contrast, we showed that the dietary inflammatory index was positively correlated with weight regain, confirming that inflammation may play a role in the regulation of body weight after weight loss. Intake of several individual micronutrients with anti-inflammatory properties, such as magnesium, folate and riboflavin, were found to be negatively correlated with weight regain.

During the weight stable period, all subjects received dietary counseling to promote a healthy eating pattern based on the recommendations of the Netherlands nutrition centre [24]. According to this guideline, individuals should increase consumption of fruits, vegetables, whole grain cereals, and fatty fish. Those types of food provide a significant amount of dietary vitamins (such as thiamin, riboflavin and folate), minerals (such as magnesium and zinc), fiber and omega-3 fatty acids, which are considered as anti-inflammatory nutrients [20,28,29]. Reduction of the consumption of saturated fat and trans fat is also recommended, which will lower the dietary inflammatory index as well [20]. By adhering to these dietary guidelines, subjects should benefit from having a diet with lower dietary inflammatory index. Except for omega-3 fatty acid intake, intake of all the above nutrients showed significant changes in the recommended direction during the weight maintenance period.

Energy intake during the weight maintenance period, but not dietary macronutrient composition, was correlated with weight regain. This finding is in line with Sacks et al. [15] who showed that the long-term maintenance of weight loss depended on the reduced calorie intake irrespective of diet composition. However, this was different from data from the DiOGenes trial [10,11] and other smaller scale intervention studies [12,13,14], which showed that increased protein intake helps to maintain weight loss. In the present study, the only dietary advice given during the WM period was to follow the Dutch national recommendations, and thus, experimental manipulation of the diet composition was minor, resulting in a wide variation of dietary compositions in a relatively limited number of subjects, making it difficult to measure effects of individual macronutrients. 

In this study, we found significant associations between the dietary inflammatory index and several of its components and weight regain. Based on our previous findings showing that weight regain was associated with continued weight-loss-induced adipocyte stress and inflammation [17,18], the hypothesis of this study was that a diet with a higher inflammatory index might also be associated with weight regain. The current results, indeed, seem to support this hypothesis. This is also supported by another study which showed that higher levels of inflammation markers in the systemic circulation (insulin, IL-6 and leukocyte number) and a higher adipose tissue inflammation were associated with resistance towards weight loss and proneness to weight regain [30]. The association between the dietary inflammatory index and obesity was previously reported in a cross-sectional study of adults [31]. This was confirmed by a large cohort study which showed that dietary inflammatory index can be used as a predictor of weight gain over eight years of follow-up, as well as of predisposition to the early development of obesity [32]. To our knowledge, this is the first study to show the effect of the dietary inflammatory index on weight regain in a weight loss trial. Overall, these results point to an important role of inflammation in the modulation of body weight, the development of obesity and the tendency for body weight regain after weight loss.

The dietary inflammatory index was based on a compilation of effects of specific nutrients and bioactive components that are derived from dietary histories. This calculation was proposed by Shivappa et al. [20] and modified by Tabung et al. [26], The positive effects of pro-inflammatory nutrients such as vitamin B12, carbohydrate, cholesterol, energy, total fat, iron, protein, saturated fat and trans fat were added up, and then the negative effects of anti-inflammatory nutrients such as vitamin B6, fiber, folic acid, Mg, MUFA, niacin, omega-3 fatty acid, omega-6 fatty acid, PUFA, riboflavin, selenium, thiamin, vitamin A, vitamin C, vitamin D and vitamin E were subtracted. One limitation of our study is that we could not include all 45 components of the dietary inflammatory index that were proposed by Shivappa et al. [20]. This has also been the case in other studies, where the number of included dietary components ranged between 17 and 44 [21,22,26,32]. 

The components of dietary inflammatory index might also have an individual impact on weight regain. Therefore, correlation analyses were done separately based on each component. We showed that the intake of several micronutrients with anti-inflammatory properties, such as magnesium, riboflavin and folate was negatively correlated with weight regain. However, from those three micronutrients, only riboflavin intake remained significantly correlated with weight regain after correction for physical activity. Regular physical activity has been shown to have anti-inflammatory properties [33]. 

There are no prior data on the association between dietary magnesium, folate and riboflavin and the protection against weight regain in overweight and obese individuals. We suggest that this effect is due to the anti-inflammatory properties of those micronutrients. Magnesium is suggested as a protective factor against oxidative stress, since hypomagnesemia was accompanied by a greater degree of oxidative stress in humans [34]. Folate has been associated with a reduction of inflammatory signals in overweight individuals [35]. Additionally, a low folate concentration was correlated with increasing risk of obesity [36]. These results suggest that there may be a potential role for magnesium and folate intake in body weight regulation via their effect on inflammation.

A role for riboflavin in the regulation of body weight has not yet been established or understood. It was previously shown that dietary riboflavin was protective against obesity [36]. In contrast, a population-based ecological study showed that a higher consumption of riboflavin per capita was associated with increased risk for obesity [37]. In vitro, induction of riboflavin in adipocytes was able to reduce pro-inflammatory factors such as tumor necrosis factor alpha, interleukin-6, MCDP-1 and HMGB1, while at the same time increasing anti-inflammatory markers such as adiponectin and interleukin 10. In addition, riboflavin supplementation in vitro was able to prevent macrophage infiltration in adipose tissue [38]. Riboflavin deficiency was associated with higher cellular stress in adipocytes, as shown by increased obesity-related apoptosis, reactive oxygen species and inflammation markers [39]. A high dietary intake of riboflavin might therefore be hypothesized to reduce weight loss-induced adipose tissue stress and inflammation and thus prevent weight regain.

This study has several strengths and limitations. To evaluate the association between dietary intake and weight regain after weight loss, this study applied an adequate study design with rigorous and strict weight loss regimes as well as a well-managed monitoring system. Compared to other long-term lifestyle-based weight loss studies, the drop-out rate of the subjects in this study was low. Limitations of this study include the small number of participants and the data collection on dietary intake for which we relied on three days of self-reported dietary records, which are known to be biased by underreporting. Underreporting of total energy intake and specific nutrients, such as saturated fat and sugar, will influence the calculation of the DII. However, this may be less important for the correlation analyses, if underreporting is comparable in all participants. Another limitation of the study is that the DII was formulated based on a limited number of items, and thus, the effect of other items in the DII will be underestimated in this study. The DII explained only ~9% of the variation in weight regain in our study. However, very strong associations are not to be expected given the complexity of body weight regulation. Several other factors, including a reduction in basal and activity-related energy expenditure, a higher level of physical activity, changes in hunger- and satiety-associated hormones, and an increase in fat cell stress in response to fat mass reduction have also been suggested to play a role [7,8,23]. Moreover, a causal relationship between DII and weight regain cannot be derived from this study. To confirm these results, further studies are needed to evaluate the effect of diets with lower inflammatory index on weight regain in an experimental design. 

## 5. Conclusions

In summary, we found no evidence for a role of macronutrient composition of diet after a weight loss intervention for the prevention of weight regain in this study. On the other hand, the inflammatory properties of the diet during the weight maintenance period may play a role in weight regain after a diet-induced weight loss program in overweight and obese adults. Further research should investigate whether tailoring diet with the aim to reduce the dietary inflammatory index is a potential approach to improve weight maintenance.

## Figures and Tables

**Table 1:** Changes in anthropometric data throughout the weight loss intervention.

	Baseline (M1)	Weight Loss Period (M2)	Weight Stable Period (M3)	Follow Up Period (M4)
Age (years)	51.3 ± 9.1			
Height (cm)	172.3 ± 8.9			
Weight (kg)	92.5 ± 9.9	83.9 ± 1.2 ***	83.7 ± 9.5	88.3 ± 10.2 ***
BMI (kg/m^2^)	31.2 ± 2.3	28.3 ± 0.3 ***	28.2 ± 2.4	29.6 ± 2.7 ***
Fat mass (%)	39.8 ± 8.6	34.8 ± 1.4 ***	33.8 ± 10.3 ***	36.3 ± 9.8 ***
Fat free mass (%)	60.2 ± 8.6	65.3 ± 1.4 ***	66.2 ± 10.3 ***	63.7 ± 9.8 ***
Fat mass (kg)	36.4 ± 7.8	28.9 ± 1.2 ***	27.9 ± 8.8 ***	31.7 ± 9.0 ***
Fat free mass (kg)	55.7 ± 11.5	54.6 ± 1.5 ***	55.2 ± 11.6 ***	56.0 ± 11.6 *
Waist circumference (cm)	102.3 ± 9.6	94.7 ± 1.2 ***	94.63 ± 9.0	98.1 ± 9.2 ***
Hip circumference (cm)	111.0 ± 6.2	105.5 ± 0.9 ***	104.7 ± 6.3 *	106.2 ± 8.3 **

* *p* < 0.05, ** *p* < 0.01, *** *p* < 0.01 paired sample *t*-test, change from M1 vs. M2, M2 vs. M3, and M3 vs. M4. Anthropometric measurements M1: before intervention; M2: end of weight loss period; M3: end of weight stable period; M4: end of follow up period.

**Table 2:** Dietary intake and physical activity at baseline and during weight maintenance in the LCD and VLCD groups and both groups combined (All).

	Baseline Diet (D1)			Weight Maintenance (D2 and D3)			
	LCD	VLCD	All	LCD	VLCD	All	*p* *
Energy (kcal/day)	1991 ± 116	2120 ± 99	2071 ± 82	1677 ± 96	1713 ± 88	1684 ± 65	<0.001
Protein (%) ^a^	17.5 ± 0.6	17.7 ± 0.9	17.6 ± 0.6	19.7 ± 0.8	20.1 ± 0.8	20.1 ± 0.6	<0.001 ^c^
Carbohydrate (%) ^a^	43.3 ± 1.3	45.0 ± 1.5	43.9 ± 7.3	48.0 ± 1.3	45.8 ± 1.1	46.8 ± 6.2	0.012
Sugars (%) ^a^	19.8 ± 1.3	20.3 ± 1.2	20.2 ± 6.5	21.6 ± 1.2	19.6 ± 1.1	20.4 ± 6.3	0.801
Fat (%) ^a^	37.0 ± 1.3	35.1 ± 1.3	36.3 ± 6.8	30.2 ± 1.0	32.3 ± 1.1	31.7 ± 5.4	<0.001
Saturated fat (g/1000 kcal) ^b^	15.2 ± 0.8	14.6 ± 0.8	15.1 ± 0.6	11.7 ± 0.5	12.7 ± 0.4	12.2 ± 0.4	<0.001
Trans fat (g/1000 kcal) ^b^	1.17 ± 0.09	1.00 ± 0.08	1.08 ± 0.06	0.85 ± 0.13	0.95 ± 0.07	0.91 ± 0.08	0.041 ^c^
MUFA (mg/1000 kcal) ^b^	13.8 ± 0.6	12.9 ± 0.5	13.5 ± 0.4	12.4 ± 0.7	12.4 ± 0.4	12.4 ± 0.4	0.104 ^c^
PUFA (mg/1000 kcal) ^b^	7.75 ±0.51	7.67 ± 0.44	7.73 ± 0.34	6.85 ± 0.35	7.20 ± 0.36	7.05 ± 0.25	0.034 ^c^
Omega 3 fatty acids (mg/1000 kcal) ^b^	0.89 ± 0.09	0.75 ± 0.08	0.83 ± 0.06	0.90 ± 0.09	0.84 ± 0.06	0.87 ± 0.06	0.584 ^c^
Omega 6 fatty acids (mg/1000 kcal) ^b^	6.04 ±0.52	6.26 ± 0.43	6.15 ± 0.35	5.27 ± 0.28	5.65 ± 0.33	5.48 ± 0.21	0.038
Cholesterol (mg/1000 kcal) ^b^	118.1 ±7.2	110.7 ± 8.1	117.2 ± 5.6	108.1 ± 8.4	106.3 ± 8.9	107.5 ± 6.1	0.202
Fiber (g/1000 kcal) ^b^	9.77 ± 0.70	11.03 ± 0.64	10.48 ± 0.50	13.28 ± 0.55	13.88 ± 0.62	13.56 ± 0.42	<0.001
Alcohol (g/1000 kcal) ^b^	3.17 ± 1.02	2.82 ± 1.04	3.12 ± 0.77	2.25 ± 0.59	2.30 ± 0.72	2.19 ± 0.46	0.185 ^c^
Magnesium (mg/1000 kcal) ^b^	145.8 ± 7.84	157.3 ± 6.1	152.2 ± 5.3	189.2 ± 6.7	182.3 ± 5.8	185.6 ± 4.6	<0.001
Iron (mg/1000 kcal) ^b^	6.14 ± 0.39	5.89 ± 0.31	6.04 ± 0.26	7.16 ± 0.33	6.94 ± 0.21	7.07 ± 0.20	0.001 ^c^
Selenium (mg/1000 kcal) ^b^	27.8 ± 2.4	25.6 ± 2.0	26.8 ± 1.7	33.3 ± 2.4	28.6 ± 2.1	31.1 ± 1.6	0.037 ^c^
Zinc (mg/1000 kcal) ^b^	5.00 ± 0.22	5.33 ± 0.34	5.19 ± 0.21	5.88 ± 0.23	5.78 ± 0.31	5.86 ± 0.2	0.022 ^c^
Vitamin A (μg/1000 kcal) ^b^	362.9 ± 34.4	360.6 ± 41.5	373.2 ± 27.7	375.2 ± 28.2	534.6 ± 77.7	449.5 ± 40.9	0.046 ^c^
Vitamin D (mg/1000 kcal) ^b^	2.31 ± 0.42	1.77 ± 0.18	2.07 ± 0.25	1.99 ± 0.18	2.11 ± 0.25	2.05 ± 0.15	0.362 ^c^
Vitamin E (mg/1000 kcal) ^b^	6.58 ± 0.52	6.89 ± 0.49	6.79 ± 0.37	6.83 ± 0.39	6.48 ± 0.48	6.65 ± 0.31	0.667
Thiamin (mg/1000 kcal) ^b^	0.64 ± 0.04	0.73 ± 0.06	0.69 ± 0.04	0.84 ± 0.05	0.72 ± 0.05	0.79 ± 0.04	0.037 ^c^
Riboflavin (mg/1000 kcal) ^b^	0.79 ± 0.07	0.78 ± 0.06	0.79 ± 0.04	0.99 ± 0.06	0.92 ± 0.07	0.97 ± 0.05	0.003 ^c^
Vitamin B6 (mg/1000 kcal) ^b^	0.89 ± 0.05	1.07 ± 0.08	0.99 ± 0.05	1.30 ± 0.08	1.25 ± 0.08	1.27 ± 0.06	<0.001 ^c^
Folate (μg/1000 kcal) ^b^	87.1 ± 10.3	95.8 ± 6.3	93.0 ± 6.4	128.1 ± 9.6	110.1 ± 6.9	119.7 ± 6.3	<0.001 ^c^
Vitamin B12 (mg/1000 kcal) ^b^	3.46 ± 0.96	2.26 ± 0.29	2.96 ± 0.55	3.27 ± 0.62	2.87 ± 0.44	3.04 ± 0.39	0.257 ^c^
Niacin (mg/1000 kcal) ^b^	9.28 ± 0.53	9.13 ± 0.61	9.15 ± 0.42	11.67 ± 0.68	10.81 ± 0.58	11.24 ± 0.46	0.001 ^c^
Vitamin C (mg/1000 kcal) ^b^	46.2 ± 7.6	54.0 ± 5.4	52.0 ± 4.9	67.4 ± 6.6	57.2 ± 5.5	61.2 ±4.2	0.025 ^c^
Dietary Inflammatory Index ^	6.11 ± 0.43	5.73 ± 0.39	5.84 ± 0.31	4.44 ± 0.35	4.49 ± 0.35	4.48 ± 0.25	<0.001 ^c^
Physical Activity ^#^	9.07 ± 0.18	9.07 ± 0.21	8.95 ± 0.14	9.06 ± 0.20	8.78 ± 0.18	9.03 ± 0.14	0.437

^a^ Dietary intake as % of total energy intake; ^b^ dietary intake in gram or milligram per 1000 kcal of total energy intake; ^c^ wilcoxon signed ranks *t*-test for non-normally distributed data. * *p* value of paired *t*-test comparing baseline diet and weight maintenance diet in the whole group. ^ Dietary inflammatory index: The sum of dietary inflammatory scores of each nutrient as calculated by Tabung et al. [26]; ^#^ physical activity was calculated using the Baecke questionnaire for habitual physical activity [27]. Data on dietary intake during the weight maintenance period are the average of dietary records obtained during the weight stable (WS) and the follow up (FU) periods; dietary intakes during WS and FU are provided in supplement 2; no significant differences were found between LCD and VLCD groups (independent *t*-test) before and during weight maintenance; LCD: low calorie diet group; VLCD: very low calorie diet group, MUFA: mono-unsaturated fatty acid; PUFA: poly-unsaturated fatty acid.

**Table 3:** Correlations between the dietary macronutrient content, the dietary inflammatory index and its components, and weight regain.

	Uncorrected Bivariate Correlation		Partial Correlation (Corrected for Physical Activity)	
	*r*	*p*	*r*	*p*
Dietary Inflammatory Index ^	0.304 ^b^	0.032 *	0.287	0.045 *
Energy ^#,^^^	0.363 ^a^	0.018 *	0.344	0.027 *
Protein ^^	−0.204 ^b^	0.196	−0.212	0.183
Carbohydrate ^^	0.157 ^a^	0.322	0.169	0.292
Sugars	0.043	0.786	0.058	0.721
Fat ^^	−0.054 ^a^	0.735	−0.106	0.510
Saturated fat ^^	−0.035 ^a^	0.824	−0.068	0.674
Trans fat ^^	−0.060 ^b^	0.704	−0.090	0.577
MUFA ^^	−0.018 ^a^	0.909	−0.059	0.714
PUFA ^^	−0.043 ^a^	0.789	−0.070	0.663
Omega 3 fatty acids ^^	−0.095 ^a^	0.551	−0.123	0.443
Omega 6 fatty acids ^^	−0.027 ^a^	0.868	−0.065	0.684
Cholesterol ^^	−0.052 ^a^	0.743	−0.060	0.711
Fiber ^^	−0.240 ^a^	0.094	−0.170	0.242
Alcohol ^^	0.103 ^b^	0.517	0.124	0.442
Magnesium ^^	−0.328 ^a^	0.034 *	−0.279	0.077
Iron ^^	−0.017 ^b^	0.916	−0.092	0.566
Selenium ^^	−0.280 ^a^	0.072	−0.277	0.080
Zinc ^^	−0.289 ^a^	0.064	−0.253	0.111
Vitamin A ^^	0.002 ^b^	0.990	−0.072	0.655
Vitamin D ^^	−0.026 ^b^	0.873	0.025	0.878
Vitamin E ^^	−0.170 ^b^	0.283	−0.273	0.084
Thiamin ^^	−0.199 ^b^	0.207	−0.138	0.388
Riboflavin ^^	−0.387 ^a^	0.011 *	−0.378	0.015 *
Vitamin B6 ^^	−0.229 ^a^	0.144	−0.206	0.197
Folate ^^	−0.313 ^b^	0.044 *	−0.290	0.066
Vitamin B12 ^^	−0.125 ^b^	0.429	−0.069	0.667
Niacin ^^	−0.130 ^a^	0.413	−0.118	0.463
Vitamin C ^^	−0.230 ^b^	0.142	−0.232	0.144

^a^ Pearson correlation for normally distributed data; ^b^ spearman correlation for non-normally distributed data; * *p* < 0.05 (2-tailed); all nutrient intakes were expressed as the total amount corrected for energy intake per day. ^#^Energy intake was expressed in kcal/day; data on dietary intake after weight loss is a combination on dietary intake during weight stable and follow up period; ^ dietary inflammatory index: The sum of dietary inflammatory scores of each nutrient as calculated by Tabung et al. [26]; ^^ Components of the dietary inflammatory index; MUFA: mono-unsaturated fatty acid; PUFA: poly-unsaturated fatty acid. Data on dietary macronutrient and DII were collected during weight maintenance period (average of D2 and D3), while weight regain was calculated as the difference in weight between follow up (M4) and weight stable (M3).

## Supplementary Materials

The following are available online at www.mdpi.com/2072-6643/9/11/1205/s1.

## References

[1] Afshin A., Forouzanfar M.H., Reitsma M.B., Sur P., Estep K., Lee A., Marczak L., Mokdad A.H., Moradi-Lakeh M., GBD 2015 Obesity Collaborators (2017). Health Effects of Overweight and Obesity in 195 Countries over 25 Years. N. Engl. J. Med..

[2] Tremmel M., Gerdtham U.-G., Nilsson P., Saha S. (2017). Economic Burden of Obesity: A Systematic Literature Review. Int. J. Environ. Res. Public Health.

[3] Ross R. (2009). The challenge of obesity treatment: Avoiding weight regain. CMAJ.

[4] Weiss E.C., Galuska D.A., Kettel Khan L., Gillespie C., Serdula M.K. (2007). Weight Regain in U.S. Adults Who Experienced Substantial Weight Loss, 1999–2002. Am. J. Prev. Med..

[5] Turk M.W., Yang K., Hravnak M., Sereika S.M., Ewing L.J., Burke L.E. (2009). Randomized clinical trials of weight-loss maintenance: A review. J. Cardiovasc. Nurs..

[6] Kraschnewski J.L., Boan J., Esposito J., Sherwood N.E., Lehman E.B., Kephart D.K., Sciamanna C.N. (2010). Long-term weight loss maintenance in the United States. Int. J. Obes..

[7] Greenway F.L. (2015). Physiological adaptations to weight loss and factors favouring weight regain. Int. J. Obes..

[8] Mariman E.C.M. (2012). Human biology of weight maintenance after weight loss. J. Nutrigenet. Nutrigenom..

[9] Elfhag K., Rössner S. (2005). Who succeeds in maintaining weight loss? A conceptual review of factors associated with weight loss maintenance and weight regain. Obes. Rev..

[10] Aller E.E.J.G., Larsen T.M., Claus H., Lindroos A.K., Kafatos A., Pfeiffer A., Martinez J.A., Handjieva-Darlenska T., Kunesova M., Stender S. (2014). Weight loss maintenance in overweight subjects on ad libitum diets with high or low protein content and glycemic index: The DIOGENES trial 12-month results. Int. J. Obes..

[11] Larsen T.M., Dalskov S.-M., van Baak M., Jebb S.A., Papadaki A., Pfeiffer A.F.H., Martinez J.A., Handjieva-Darlenska T., Kunešová M., Pihlsgård M. (2010). Diets with high or low protein content and glycemic index for weight-loss maintenance. N. Engl. J. Med..

[12] Claessens M., van Baak M.A., Monsheimer S., Saris W.H.M. (2009). The effect of a low-fat, high-protein or high-carbohydrate *ad libitum* diet on weight loss maintenance and metabolic risk factors. Int. J. Obes..

[13] Westerterp-Plantenga M.S., Lejeune M.P.G.M., Nijs I., van Ooijen M., Kovacs E.M.R. (2004). High protein intake sustains weight maintenance after body weight loss in humans. Int. J. Obes..

[14] Hursel R., Westerterp-Plantenga M.S. (2009). Green tea catechin plus caffeine supplementation to a high-protein diet has no additional effect on body weight maintenance after weight loss. Am. J. Clin. Nutr..

[15] Sacks F.M., Bray G.A., Carey V.J., Smith S.R., Ryan D.H., Anton S.D., McManus K., Champagne C.M., Bishop L.M., Laranjo N. (2009). Comparison of Weight-Loss Diets with Different Compositions of Fat, Protein, and Carbohydrates. N. Engl. J. Med..

[16] Kjølbæk L., Sørensen L.B., Søndertoft N.B., Rasmussen C.K., Lorenzen J.K., Serena A., Astrup A., Larsen L. (2017). Protein supplements after weight loss do not improve weight maintenance compared with recommended dietary protein intake despite beneficial effects on appetite sensation and energy expenditure: A randomized, controlled, double-blinded trial. Am. J. Clin. Nutr..

[17] Roumans N.J.T., Vink R.G., Fazelzadeh P., Van Baak M.A., Mariman E.C.M. (2017). A role for leukocyte integrins and extracellular matrix remodeling of adipose tissue in the risk of weight regain after weight loss. Am. J. Clin. Nutr..

[18] Roumans N.J.T., Camps S.G., Renes J., Bouwman F.G., Westerterp K.R., Mariman E.C.M. (2016). Weight loss-induced stress in subcutaneous adipose tissue is related to weight regain. Br. J. Nutr..

[19] Cavicchia P.P., Steck S.E., Hurley T.G., Hussey J.R., Ma Y., Ockene I.S., Hebert J.R. (2009). A New Dietary Inflammatory Index Predicts Interval Changes in Serum High-Sensitivity C-Reactive Protein. J. Nutr..

[20] Shivappa N., Steck S.E., Hurley T.G., Hussey J.R., Hébert J.R. (2014). Designing and developing a literature-derived, population-based dietary inflammatory index. Public Health Nutr..

[21] Shivappa N., Steck S.E., Hurley T.G., Hussey J.R., Ma Y., Ockene I.S., Tabung F., Hébert J.R. (2014). A population-based dietary inflammatory index predicts levels of C-reactive protein in the Seasonal Variation of Blood Cholesterol Study (SEASONS). Public Health Nutr..

[22] Shivappa N., Hébert J.R., Rietzschel E.R., De Buyzere M.L., Langlois M., Debruyne E., Marcos A., Huybrechts I. (2015). Associations between dietary inflammatory index and inflammatory markers in the Asklepios Study. Br. J. Nutr..

[23] Vink R.G., Roumans N.J.T., Arkenbosch L.A.J., Mariman E.C.M., Van Baak M.A. (2016). The effect of rate of weight loss on long-term weight regain in adults with overweight and obesity. Obesity.

[24] Netherlands Nutrition Center Richtlijnen Voedselkeuze. http://www.voedingscentrum.nl/professionals/schijf-van-vijf/Richtlijnen.aspx.

[25] Willett W., Stampfer M.J. (1986). Total energy intake: Implications for epidemiologic analyses. Am. J. Epidemiol..

[26] Tabung F.K., Smith-Warner S.A., Chavarro J.E., Fung T.T., Hu F.B., Willett W.C., Giovannucci E.L. (2017). An Empirical Dietary Inflammatory Pattern Score Enhances Prediction of Circulating Inflammatory Biomarkers in Adults. J. Nutr..

[27] Baecke J.A.H., Burema J., Frijters J.E.R. (1982). A short questionnaire for the measurement of habitual physical activity in epidemiological studies. Am. J. Clin. Nutr..

[28] Vicente A.R., Manganaris G.A., Sozzi G.O., Crisosto C.H. (2009). Nutritional Quality of Fruits and Vegetables. Postharvest Handling.

[29] Gebauer S.K., Psota T.L., Harris W.S., Kris-Etherton P.M. (2006). n-3 Fatty acid dietary recommendations and food sources to achieve essentiality and cardiovascular benefits. Am. J. Clin. Nutr..

[30] Kong L.C., Wuillemin P.-H., Bastard J.-P., Sokolovska N., Gougis S., Fellahi S., Darakhshan F., Bonnefont-Rousselot D., Bittar R., Doré J. (2013). Insulin resistance and inflammation predict kinetic body weight changes in response to dietary weight loss and maintenance in overweight and obese subjects by using a Bayesian network approach. Am. J. Clin. Nutr..

[31] Ruiz-Canela M., Zazpe I., Shivappa N., Hébert J.R., Sánchez-Tainta A., Corella D., Salas-Salvadó J., Fitó M., Lamuela-Raventós R.M., Rekondo J. (2015). Dietary inflammatory index and anthropometric measures of obesity in a population sample at high cardiovascular risk from the PREDIMED (PREvención con DIeta MEDiterránea) trial. Br. J. Nutr..

[32] Ramallal R., Toledo E., Martínez J.A., Shivappa N., Hébert J.R., Martínez-González M.A., Ruiz-Canela M. (2017). Inflammatory Potential of Diet, Weight Gain, and Incidence of Overweight/Obesity: The SUN Cohort. Obesity.

[33] Gleeson M., Bishop N.C., Stensel D.J., Lindley M.R., Mastana S.S., Nimmo M.A. (2011). The anti-inflammatory effects of exercise: Mechanisms and implications for the prevention and treatment of disease. Nat. Rev. Immunol..

[34] Morais J.B.S., Severo J.S., Santos L.R.D., de Sousa Melo S.R., de Oliveira Santos R., de Oliveira A.R.S., Cruz K.J.C., do Nascimento Marreiro D. (2017). Role of Magnesium in Oxidative Stress in Individuals with Obesity. Biol. Trace Elem. Res..

[35] Solini A., Santini E., Ferrannini E. (2006). Effect of short-term folic acid supplementation on insulin sensitivity and inflammatory markers in overweight subjects. Int. J. Obes..

[36] Gunanti I.R., Marooks G.C., Al-Mamun A., Long K.Z. (2014). Low serum vitamin B-12 and folate concentrations and low thiamin and riboflavin intakes are inversely associated with greater adiposity in Mexican American children. J. Nutr..

[37] Zhou S.-S., Li D., Zhou Y.-M., Sun W.-P., Liu Q.-G. (2010). B-vitamin consumption and the prevalence of diabetes and obesity among the US adults: Population based ecological study. BMC Public Health.

[38] Mazur-Bialy A.I., Pocheć E. (2016). Riboflavin reduces pro-inflammatory activation of adipocyte-macrophage co-culture. Potential application of vitamin B2 enrichment for attenuation of insulin resistance and metabolic syndrome development. Molecules.

[39] Mazur-Bialy A.I., Pocheć E. (2017). Vitamin B2 deficiency enhances the pro-inflammatory activity of adipocyte, consequences for insulin resistance and metabolic syndrome development. Life Sci..

