# Supplementary material for: Dietary Intake after Weight Loss and the Risk of Weight Regain: Macronutrient Composition and Inflammatory Properties of the Diet
Source: Nutrients. 2017 Nov 2;9(11):1205. doi: 10.3390/nu9111205 (PMC5707677; doi:10.3390/nu9111205)
Supplement: Supplementary file 1 [file nutrients-09-01205-s001.docx]

**Supplement 1.** Anthropometric data throughout the weight loss intervention.

|  | **Baseline**  **(M1)** | | **Weight Loss**  **Period (M2)** | | **Weight Stable**  **Period (M3)** | | **Follow Up**  **Period (M4)** | |
| --- | --- | --- | --- | --- | --- | --- | --- | --- |
|  | **LCD**  **(n=29)** | **VLCD**  **(n=28)** | **LCD (n=29)** | **VLCD (n=28)** | **LCD (n=29)** | **VLCD (n=28)** | **LCD (n=28)** | **VLCD (n=27)** |
| Age (years) | 51.8 ± 1.9 | 50.7 ± 1.5 |  |  |  |  |  |  |
| Height (cm) | 171.7 ± 1.6 | 172.9 ± 1.8 |  |  |  |  |  |  |
| Weight (kg) | 92.4 ± 1.9 | 92.6 ± 1.8 | 84.2 ± 1.9 | 83.6 ± 1.6 | 84.0 ± 1.9 | 83.3 ± 1.7 | 88.4 ± 2.0 | 88.1 ± 1.9 |
| BMI (kg/m^2^) | 31.3 ± 0.5 | 31.0 ± 0.4 | 28.6 ± 0.5 | 28.0 ± 0.4 | 28.5 ± 0.5 | 27.9 ± 0.4 | 30.0 ± 0.5 | 29.2 ± 0.5 |
| Fat mass (%) | 39.9 ± 1.8 | 39.7 ± 1.5 | 34.5 ± 2.1 | 35.0 ± 1.8 | 33.8 ± 2.1 | 33.8 ± 1.8 | 36.5 ± 2.0 | 36.1 ± 1.8 |
| Fat free mass (%) | 60.1 ± 1.8 | 60.3 ± 1.5 | 65.5 ± 2.1 | 65.0 ± 1.8 | 66.2 ± 2.1 | 66.2 ± 1.8 | 63.5 ± 2.0 | 63.9 ± 1.8 |
| Fat mass (kg) | 36.6 ± 1.7 | 36.2 ± 1.2 | 29.0 ± 1.9 | 28.8 ± 1.4 | 28.3 ± 2.0 | 27.5 ± 1.3 | 32.2 ± 2.0 | 31.3 ± 1.5 |
| Fat free mass (kg) | 55.4 ± 2.2 | 55.9 ± 2.2 | 54.9 ± 2.1 | 54.3 ± 2.2 | 55.2 ± 2.2 | 55.1 ± 2.3 | 56.0 ± 2.3 | 56.1 ± 2.3 |
| Waist circumference (cm) | 102.6 ± 2.0 | 101.9 ± 1.5 | 95.3 ± 1.8 | 94.2 ± 1.4 | 94.3 ± 2.0 | 95.0 ± 1.3 | 98.3 ± 1.9 | 97.9 ± 1.6 |
| Hip circumference (cm) | 110.8 ± 1.3 | 111.1 ± 1.1 | 105.8 ± 1.4 | 105.2 ± 1.0 | 104.6 ± 1.4 | 104.9 ± 0.9 | 106.7 ± 1.8 | 105.7 ± 1.3 |

No significant differences were found between LCD and VLCD at any time point.

Supplement 2. Data on dietary intake, dietary inflammatory index, and physical activity at D2 and D3

|  | **Weight Stable**  **(D2)** | | | **Follow Up**  **(D3)** | | |
| --- | --- | --- | --- | --- | --- | --- |
|  | **LCD** | **VLCD** | **All** | **LCD** | **VLCD** | **All** |
| Energy intake (kcal/day) | 1666 ± 89 | 1614 ± 91 | 1641 ± 64 | 1773 ± 147 | 1827 ± 100 | 1800 ± 88 |
| Protein (%)^a^ | 21.9 ± 1.0 | 20.5 ± 0.8 | 21.2 ± 0.6 | 18.2 ± 1.0 | 19.4 ± 0.8 | 18.8 ± 0.6^##^ |
| Carbohydrate (%)^a^ | 46.6 ± 1.6 | 47.2 ± 0.9 | 46.9 ± 0.9 | 48.5 ± 1.4 | 45.3 ± 1.6 | 46.9 ± 1.1 |
| Sugars (%)^a^ | 22.1 ± 1.5 | 19.3 ± 1.2 | 20.8 ± 1.0 | 12.9 ± 1.1 | 11.06 ± 0.77 | 11.97 ± 0.69^###^ |
| Fat (%)^a^ | 30.2 ± 1.3 | 31.0 ± 1.0 | 30.7 ± 0.8 | 30.9 ± 1.2 | 33.2 ± 1.5 | 32.1 ± 0.9 |
| Saturated fat (g/ 1000 kcal)^b^ | 11.6 ± 0.6 | 11.9 ± 0.5 | 11.7 ± 0.4 | 11.9 ± 0.8 | 13.7 ± 0.8 | 12.8 ± 0.6 |
| Trans fat (g/ 1000 kcal)^b^ | 0.87 ± 0.15 | 0.90 ± 0.07 | 0.89 ± 0.09 | 0.79 ± 0.09 | 1.00 ± 0.10 | 0.89 ± 0.068 |
| MUFA (mg/ 1000 kcal)^b^ | 12.2 ± 0.8 | 11.7 ± 0.5 | 11.9 ± 0.5 | 11.9 ± 0.5 | 12.8 ± 0.61 | 12.3 ± 0.4 |
| PUFA (mg/ 1000 kcal)^b^ | 6.36 ± 0.37 | 7.40 ± 0.45 | 6.85 ± 0.30 | 7.15 ± 0.45 | 6.66 ± 0.54 | 6.91 ± 0.35 |
| Omega 3 fatty acids (mg/ 1000 kcal)^b^ | 0.88 ± 0.08 | 0.83 ± 0.07 | 0.86 ± 0.05 | 0.91 ± 0.14 | 0.85 ± 0.10 | 0.88 ± 0.08 |
| Omega 6 fatty acids (mg/ 1000 kcal)^b^ | 4.98 ± 0.30 | 5.79 ± 0.44 | 5.36 ± 0.26 | 5.36 ± 0.37 | 5.27 ± 0.45 | 5.31 ± 0.29 |
| Cholesterol (mg/ 1000 kcal)^b^ | 116.6 ± 12.1 | 96.5 ± 9.1 | 107.2 ± 7.8 | 98.72 ± 10.01 | 115.5 ± 12.9 | 107.1 ± 8.2 |
| Fiber (g/ 1000 kcal)^b^ | 13.82 ± 0.70 | 15.09 ± 0.93 | 14.42 ± 0.57 | 12.79 ± 0.86 | 12.40 ± 0.59 | 12.59 ± 0.52^#^ |
| Alcohol (g/ 1000 kcal)^b^ | 1.93 ± 0.66 | 1.76 ± 0.75 | 1.85 ± 0.49 | 3.26 ± 0.92 | 3.02 ± 0.93 | 3.14 ± 0.65 |
| Magnesium (mg/ 1000 kcal)^b^ | 194.9 ± 7.1 | 188.3 ± 7.1 | 191.8 ± 5.0 | 181.7 ± 10.7 | 177.9 ± 6.7 | 179.8 ± 6.2 |
| Iron (mg/ 1000 kcal)^b^ | 7.17 ± 0.32 | 7.10 ± 0.26 | 7.14 ± 0.21 | 7.31 ± 0.55 | 6.89 ± 0.21 | 7.10 ± 0.29 |
| Selenium (mg/ 1000 kcal)^b^ | 35.2 ± 2.6 | 28.6 ± 2.4 | 32.1 ± 1.8 | 28.3 ± 2.3 | 28.9 ± 2.8 | 28.6 ± 1.8 |
| Zinc (mg/ 1000 kcal)^b^ | 6.19 ± 0.23 | 6.17 ± 0.36 | 6.18 ± 0.21 | 5.45 ± 0.43 | 5.56 ± 0.30 | 5.50 ± 0.26^#^ |
| Vitamin A (μg/ 1000 kcal)^b^ | 355.5 ± 29.6 | 626.0 ± 134.2 | 483.1 ± 67.2 | 403.2 ± 44.1 | 451.0 ± 58.3 | 427.1 ± 36.3 |
| Vitamin D (mg/ 1000 kcal)^b^ | 1.88 ± 0.19 | 2.09 ± 0.18 | 1.98 ± 0.13 | 2.08 ± 0.27 | 2.19 ± 0.50 | 2.13 ± 0.28 |
| Vitamin E (mg/ 1000 kcal)^b^ | 6.45 ± 0.45 | 6.52 ± 0.59 | 6.49 ± 0.36 | 7.17 ± 0.58 | 6.60 ± 0.60 | 6.89 ± 0.42 |
| Thiamin (mg/ 1000 kcal)^b^ | 0.82 ± 0.05 | 0.80 ± 0.06 | 0.81 ± 0.04 | 0.85 ± 0.08 | 0.67 ± 0.05 | 0.76 ± 0.05 |
| Riboflavin (mg/ 1000 kcal)^b^ | 1.06 ± 0.07 | 0.97 ± 0.07 | 1.02 ± 0.05 | 0.91 ± 0.09 | 0.88 ± 0.10 | 0.89 ± 0.07 |
| Vitamin B6 (mg/ 1000 kcal)^b^ | 1.36 ± 0.09 | 1.41 ± 0.10 | 1.38 ± 0.07 | 1.25 ± 0.10 | 1.07 ± 0.08 | 1.16 ± 0.07^##^ |
| Folate (μg/ 1000 kcal)^b^ | 129.4 ± 9.6 | 115.9 ± 9.7 | 123.03 ± 6.8 | 121.4 ± 15.19 | 106.6 ± 5.1 | 114.0 ± 7.99 |
| Vitamin B12 (mg/ 1000 kcal)^b^ | 3.57 ± 0.77 | 3.06 ± 0.73 | 3.33 ± 0.53 | 2.94 ± 0.64 | 2.80 ± 0.34 | 2.86 ± 0.36 |
| Niacin (mg/ 1000 kcal)^b^ | 11.92 ± 0.76 | 10.99 ± 0.67 | 11.48 ± 0.51 | 10.84 ± 0.92 | 10.30 ± 0.69 | 10.57 ± 0.57 |
| Vitamin C (mg/ 1000 kcal)^b^ | 76.2 ± 8.1 | 57.2 ± 6.5 | 67.2 ± 5.4 | 52.5 ± 7.0 | 57.4 ± 6.3 | 54.9 ± 4.7 |
| Dietary Inflammatory Index^ | 4.37 ± 0.36 | 4.10 ± 0.44 | 4.24 ± 0.28 | 4.60 ± 0.54 | 5.14 ± 0.35 | 4.87 ± 0.32 |
| Physical Activity | 9.21 ± 0.22 | 8.97 ± 0.18 | 9.06 ± 0.14 | 8.95 ± 0.20 | 8.60 ± 0.21 | 8.80 ± 0.14^##^ |

^a^Dietary intake as % of total energy intake; ^b^Dietary intake in gram or milligram per 1000 kcal of total energy intake; ^Dietary inflammatory index: The sum of dietary inflammatory scores of each nutrient as calculated by Tabung et al [26]⁠; P value ^#^<0.05, ^##^<0.01 and ^###^<0.001 for difference between D2 and D3 (paired t-test or Wilcoxon test). No significant differences were found between LCD and VLCD groups (independent t-test) at any time point. LCD: low calorie diet group ; VLCD: very low calorie diet group, MUFA: mono-unsaturated fatty acid; PUFA: poly-unsaturated fatty acid.
